# Parent-adolescent conflict: an exploration from the perspective of Vietnamese adolescents

**DOI:** 10.3389/fpsyg.2023.1243494

**Published:** 2023-11-07

**Authors:** Thi Hong Hanh Nguyen, Thi Nhu Trang Nguyen

**Affiliations:** ^1^Department of Children and Adolescent Studies, Institute for Family and Gender Studies, Hanoi, Vietnam; ^2^Department of Social Work, Faculty of Sociology, VNU-Hanoi University of Social Sciences and Humanities, Hanoi, Vietnam

**Keywords:** parent-adolecent conflict, Vietnamese families, conflict resolution, emotional response to conflict, behavioral response to conflict, friend network

## Abstract

This research investigates parent-adolescent conflicts from the viewpoint of Vietnamese adolescents. Employing a mixed-method approach, the study conducted in-depth interviews and a questionnaire survey with 706 high-school students. The findings highlight that conflicts between Vietnamese parents and adolescents commonly arise regarding internet usage for entertainment and academic purposes. Furthermore, adolescents reported having more conflicts with their mothers than with their fathers. Although instances of parental aggression were identified, most adolescents perceived their parents’ conflict resolution as supportive. However, Vietnamese adolescents tended to display passive behavioral responses during conflicts. The research also identified certain emotional responses as warning signs of mental health issues, including suicidal thoughts, among some adolescents. Moreover, a higher frequency of conflicts with parents was significantly associated with increased peer connections. The study emphasizes the importance of professionals, such as school social workers or counselors, prioritizing the understanding of parent–child conflicts’ impact on adolescents’ emotions and mental health. Additionally, it underscores the significance of examining parenting patterns and parent–child communication within contemporary Vietnamese families.

## 1. Introduction

Conflict is an inevitable part of social relationships, and disagreements often arise between parents and children within families. During the adolescent period, parent–child conflict tends to occur more frequently and intensively, and adolescents experience more conflict with their parents compared to their peers (Van Doorn et al., 2008; Moed et al., 2015). This heightened conflict between adolescents and their parents can be attributed, in part, to the developmental drives of adolescence, which push adolescents to seek greater independence from their parents while simultaneously desiring closer connections with their peers (Branje, 2018). In addition, cognitive development during this period allows adolescents to differentiate between closed-field relationships (such as those with parents) and open-field relationships (such as those with peers) and become more aware of the vulnerability of open-field relationships to rupture when conflicts arise (Hartup, 1992). Consequently, adolescents tend to carefully manage and minimize conflict with their peers while displaying a less protective attitude when it comes to handling disagreements within family relationships.

Parent–child conflict is not only an inevitable aspect of family life but also serves as a functional component of healthy family dynamics across cultures. Conflict within the parent–child relationship has both potential risks and benefits. Conflict provides opportunities for growth and learning in the parent–child relationship. It facilitates the child’s transition from an instinctive being to a social being by helping them understand and navigate differences, social norms, conflict resolution patterns, and skills necessary for social engagement (Wrench et al., 2020). However, an excessive or inadequate amount of conflict may indicate unhealthy family dynamics, such as neglectful or highly authoritarian parenting styles. Adolescents also learn from their parents’ conflict management styles and apply these lessons in their interactions outside the family context (Van Doorn et al., 2008). Branje (2018) emphasizes that parent-adolescent conflicts can serve as mechanisms for change and development within the parent–child relationship. If parents and adolescents can effectively manage emotional variability during conflicts, these conflicts can provide opportunities for relationship development and meeting the needs of adolescents. However, if parents exhibit negative behaviors and responses during conflicts, adolescents may internalize these patterns and adopt negative strategies for conflict resolution. Research has shown that children of coercive parents are more likely to resort to coercive resolutions and are at higher risk of developing non-compliance and antisocial behaviors (DeGarmo, 2010). Excessive conflict during adolescence has been associated with maladaptive outcomes, including behavioral and emotional difficulties (Moed et al., 2015).

Indeed, the way parents and adolescents handle their conflicts has a profound influence on the psychological development of adolescents. Research has shown that the resolution of parent-adolescent conflicts has an impact on the development of adolescents’ empathy (Van Lissa et al., 2015). Specifically, the way parents handle conflicts can either enhance or hinder the development of empathy in adolescents. Studies conducted on Singaporean youths by Keng and Wong (2017) have shed light on the relationship between parental invalidation and borderline personality disorder. Parental invalidation, which is a common parenting trait observed in Asian cultures, was found to be positively associated with borderline personality disorder symptoms. Furthermore, Keng and Soh (2018) found that the mother–child relationship had a more significant impact on the child than the father-child relationship in this regard. They also highlighted that the child’s conformity mediated the association between maternal invalidation and borderline personality disorder symptoms. Considering the differential impact of mothers’ and fathers’ interactions with adolescents during conflicts, Renk et al. (2005) suggested that the examination of parent-adolescent conflicts should take into account the gender of the parent(s) involved. This aligns with the findings of Keng and Soh (2018) regarding the stronger influence of the mother–child relationship. The dynamics of parent-adolescent conflicts may vary depending on the gender of the parent, and it is essential to consider these nuances in understanding the impact of conflicts on adolescents.

Whereas this topic has been examined in many cultural contexts, there is a limited understanding of the specific dynamics and issues surrounding parent-adolescent conflicts in Vietnamese families, whereas the parent-adolescent conflict in Vietnam might be an interesting case to study. For thousand of years, the parent–child relationship in Vietnamese families has been regulated by Confucian ideology which emphasized the submission of children to their parents. The effect of Confucian ideology on family relationships, especially between parents and children, remained robust during French colonization and even when Vietnam gained independence and established socialist government. Radical changes in political regime tended to exert its impact on socio-economic aspects of Vietnam society rather than the interactions between parents and children, partly because this period Vietnam was quite a closed society due to the trade ban imposed to Vietnam by the US However, economic renovation (often known as Doi Moi) in 1986 has brought about vital changes to family functioning (Teramoto et al., 2017). Market-oriented economy encouraged women to participate in labor force as an equivalent family’s breadwinner to men, raised both men’s and women’s time and energy spent in economic sphere, and hence some of the traditional functions of family as educating children or taking care of the children and older persons must be transferred to social services. Especially since 1994 when the US lifted its 30-year trade embargo on Vietnam, Vietnam rapidly developed international affairs, which in turn let Vietnamese families and individuals exposed to international values and norms. These changes in family functioning and the adoption of international perspectives about the self and the family create growing inter-generational gap between adults and adolescents. Whereas adults tend to attach to traditional norms and values, young persons are more inclined to Western values and norms, and consider themselves as “different kind of citizens” from previous generations (Nilan, 1999: 354).

In this context, understanding parent-adolescent conflict in general and in Vietnamese families in particular is of great importance, since it helps identify the contemporary issues in families such as current concerns of and differences in major concerns between parents and adolescents, how parents and adolescents respond to each other during their conflicts, how they perceive of their roles as members of family and in relation to each other. In the long run, such understanding can help identify the changes in family as an important social institution and socialization agent for children in the context of broader social changes. Particularly, when mental health of adolescents in Vietnam is a growing concern (UNICEF, 2022), tracing back original foundation of mental health as family interactions as well as parent–child conflict may contribute significant implications for professionals as counselors and social workers when working with adolescents and their families.

## 2. Research methods

### 2.1. Research design

Because this topic has been underexplored in Vietnam, this study applied an exploratory approach to seek some insights into the conflicts between adolescents and their parents in contemporary Vietnamese families. The study used two methods: semi-structured interview and questionnaire survey. Semi-structured interviews were used to explore themes of conflicts between adolescents and their parents, the motivations, feelings and reasonings underlying the way they response to the conflict, and identify the factors associated with conflicts and conflict resolutions. In total, researchers conducted 16 semi-structured interviews with high-school students, 06 with parents, and 04 with high-school homeroom teachers. The qualitative interview sample was purposively selected. Researchers asked homeroom teachers at each school to introduce and connect some students and parents of students who often met troubles at schools, and some students and parents of students who did good academic and behavioral performance at school. All interviews were conducted in person (between a researcher and an interviewee) at the psychological counselling room at the school to ensure the safety, privacy, and comfort of interviewees. Interviews were recorded upon interviewees’ consent. Each interview usually lasted 60 min. The collected qualitative data was then analyzed using NVivo. Based on content analysis of these in-depth interviews, a self-administrated questionnaire for high-school students was designed. A pilot study was conducted with 30 students before the questionnaire was finalized.

In the second stage, the study invited students at the two high schools to participate in the questionnaire survey. There are three grades in high schools in Vietnam: 10, 11, and 12. In the urban school there are 8 classes in each grade, each class has about 50 students. In the rural school there are 7 classes in each grade, each class has about 40 students. At each school, we randomly selected two classes from each grade, using lottery method. Then we invited all students in the selected class to participate in the survey. To ensure the representativeness of the sample, we planned that the questionnaire survey would be conducted at the selected class only if (1) less than 10% of the students refuse to participate in the survey and (2) there is no typical characteristics of the students who refuse (for example, most of them have poor academic performance; or have record of maladaptive behaviors). All of the students agreed to join in the survey. In total, 706 students participated in and completed the questionnaire survey.

Before interviewing the students, we first contacted the schools, explaining the study and asking their permission to conduct the interview with their students. With the permission of the schools, we sent each student an invitation letter and a Question-and-Answer leaflet explaining the study and some other information such as possible benefits and risk of taking part in the study. Two days after sending the letter and leaflet, investigators contacted the students asking if they understood the study and agreed to participate. At the day of interviewing, consent forms were provided to the participants. Students’ right to withdraw from the study anytime they want without any harm was also reminded again before the interview started. The research design was reviewed and approved by the IRB of the Institute for Family and Gender Studies, under Decision 03/HĐKH-GĐ&G signed on 06 January 2020. The questionnaire survey sample can be summarized as in Table 1.

**Table 1:** Sample characteristics.

	*N*	Percentage
**Gender**		
Boy	285	40.4
Girl	421	59.6
**Living area**		
Rural	329	46.6
Urban	377	53.4
**Grade**		
Grade 10	242	34.3
Grade 11	249	35.3
Grade 12	215	30.5

### 2.2. Measuring parent-adolescent conflict

#### 2.2.1. Parent-adolescent specific conflict checklist

According to qualitative data analysis, 09 popular conflict themes were identified: 1-conflict over adolescents’ dressing and hair style; 2-time spent with friends, 3-academic performance, 4-taking extra-classes; 5-higher education orientation (choosing the university and the major to apply for); 6-self-study at home; 7-money management; 8-time spent on internet (either on their computer or smartphone) for entertainment purpose (including playing online games); and 9-choosing friends. In the questionnaire, each of the conflicts was measured on a 4-point Likert scale, with 0 = hardly having conflict; 1 = sometimes per month; 2 = sometimes per week; and 3 = nearly daily.

#### 2.2.2. Frequency of parent-adolescent conflict

This variable was created by computing all the 09 specific conflicts, its value was recoded into 4-point scale, with 0 = low frequency; and 3 = very high frequency.

## 3. Results

### 3.1. Adolescents’ frequency of having conflict with their parents

When being asked about the frequency of having conflict with their parents in the last three months, with 0 = hardly; 1 = sometimes a month; 2 = sometimes a week; 3 = nearly daily, results show that Vietnamese adolescents have more conflict with their mother than with father in all reasons for conflict. However, the trends of having conflict with father and mother are quite similar, as shown in Table 2.

**Table 2:** Frequency of parent-adolescent conflicts by conflict themes.

Adolescents’ conflict with their father and mother over…	*N*	Range	Father		Mother	
			Mean	SD	Mean	SD
Dressing and hair style	709	0–3	0.39	0.666	0.48	0.717
Time spent with friends	709	0–3	0.58	0.773	0.65	0.787
Academic performance	709	0–3	0.67	0.766	0.85	0.823
Extra classes	709	0–3	0.45	0.707	0.56	0.765
Higher education orientation	709	0–3	0.38	0.633	0.47	0.695
Self-study at home	709	0–3	0.82	0.926	0.99	0.959
Money management	709	0–3	0.40	0.647	0.48	0.715
Time spent on internet for entertainment	709	0–3	1.27	0.998	1.46	0.715
Choosing friend	709	0–3	0.35	0.616	0.41	0.637

As reported by adolescents, their conflicts with both father and mother occur most frequently on the amount of time they spent on internet for entertainment purpose (highest mean values at 1.27 and 1.46 respectively), following by self-study at home (0.82 and 0.99, respectively) and academic performance (0.67 and 0.85, respectively). Adolescents have least conflict with their father and mother on choosing friend (0.35 and 0.41, respectively); however, more conflicts occur on their spending time with friends (0.58 and 0.65, respectively).

To further examine if there is an intra-role conflict between mother and daughter, father and son, one-way ANOVA analysis is used to see if there is any difference in mean scores of boy group and girl group in having conflict with their father and mother. The result is presented in Table 3.

**Table 3:** Difference between boys and girls in frequency of having conflict with their father and mother, by conflict themes.

	Adolescent’s sex	*N*	Mean	SD	*F* (1, 698)	*p*-value
**Conflict with father over…**						
Dressing and hair style	Boy	279	0.43	0.695	0.988	0.320
	Girl	421	0.38	0.649		
Time spent with friends	Boy	279	0.69	0.848	8.191	0.004
	Girl	421	0.52	0.716		
Academic performance	Boy	279	0.82	0.846	17.294	0.000
	Girl	421	0.58	0.686		
Extra classes	Boy	279	0.57	0.835	13.235	0.000
	Girl	421	0.37	0.600		
Higher education orientation	Boy	279	0.47	0.731	8.613	0.003
	Girl	421	0.33	0.555		
Self-study at home	Boy	279	0.97	0.987	12.171	0.001
	Girl	421	0.72	0.874		
Money management	Boy	279	0.46	0.697	3.763	0.053
	Girl	421	0.36	0.607		
Time spent on internet for entertainment	Boy	279	1.39	0.994	6.448	0.011
	Girl	421	1.19	0.990		
Choosing friends	Boy	279	0.41	0.688	3.897	0.049
	Girl	421	0.32	0.563		
**Conflict with mother over…**						
Dressing and hair style	Boy	279	0.47	0.688	0.215	0.643
	Girl	421	0.49	0.739		
Time spent with friends	Boy	279	0.67	0.834	0.374	0.541
	Girl	421	0.64	0.758		
Academic performance	Boy	279	0.97	0.893	10.240	0.001
	Girl	421	0.77	0.769		
Extra classes	Boy	279	0.62	0.848	3.295	0.070
	Girl	421	0.52	0.699		
Higher education orientation	Boy	279	0.51	0.763	1.957	0.162
	Girl	421	0.44	0.643		
Self-study at home	Boy	279	1.08	0.971	4.33	0.038
	Girl	421	0.93	0.949		
Money management	Boy	279	0.49	0.753	0.378	0.539
	Girl	421	0.46	0.684		
Time spent on internet for entertainment	Boy	279	1.49	1.003	0.312	0.577
	Girl	421	1.44	1.005		
Choosing friends	Boy	279	0.42	0.679	0.234	0.628
	Girl	421	0.40	0.611		

According to the results, there is no difference between girls and boys in their frequency of having conflict with their mother in almost every reason for conflict, except for academic performance and self-study at home. For the two reasons for conflict, academic performance and self-study at home, mean scores show that the frequency of having conflict between mother and boy are higher than between mother and girl, and these differences are statistically significant.

About the conflicts between fathers and their boys/girls, mean scores show that fathers have more conflicts with their boys than with their girls in all reasons for conflicts; and the differences between these two groups (fathers and boys; fathers and girls) are statistically significant in most of the reasons for conflict except for adolescents’ dressing and hair style and money management.

### 3.2. How Vietnamese adolescents and their parents handle their conflict and adolescents’ responses to conflict resolution

**Figure 1:**
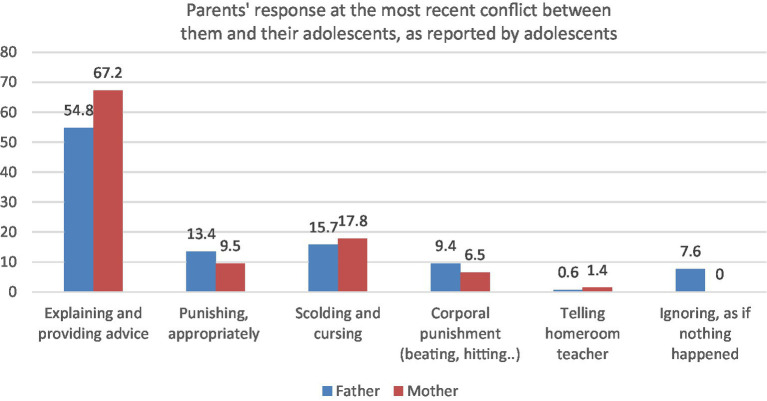
Parents’ response at the most recent conflict, as reported by adolescents.

When being asked how their mother and father respond at the most recent conflict between them, most adolescents report that their parents’ response is quite supportive, as presented in Figure 1.

Among eight parental responses against parent-adolescent conflict as documented in the study, the most supportive response – explaining the situation and providing advice – is the most popular reaction among both fathers and mothers. 54.8% of adolescent participants report that their father and 67.2% report that their mother does so in the most recent conflict between them and their parents. Lower percentage reports aggressive response from their parents, and fathers tend to have more physically aggressive responses (corporal punishment such as beating or hitting) than mothers (9.4 and 6.5% reported, respectively), whereas mothers tend to have more verbally and emotionally aggressive reaction than fathers (17.8% and 15.7%, respectively). Some parents also tell their adolescents’ homeroom teachers for their intervention into the conflict between parents and adolescents, as reported by adolescents (0.6% of fathers, 1.4% of mother). Notably, whereas 7.6% of adolescents report that their fathers ignore the conflict as if nothing happened, none of the participants report that type of reactions from their mothers.

**Figure 2:**
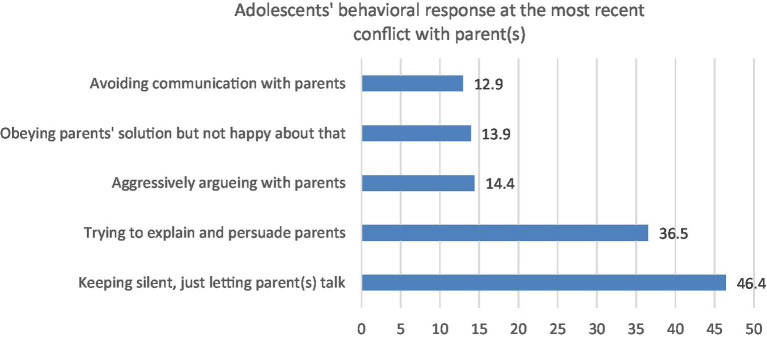
Adolescents’ response in the most recent conflict.

The following figure presents adolescents’ response to their parents during conflict (See Figure 2).

As reported by adolescents, 36.5% of them try to explain their situation to parents and persuade parents to accept or even support them in the situation. The remaining 63.5% report either passive response (46.4% kept silent, just letting their parent(s) talk; 13.9% obeyed parents’ solution even though they was unhappy about that; and 12.9% avoided communicating with their parents about the conflict) or aggressive response (14.4% aggressively agued with their parents).

**Figure 3:**
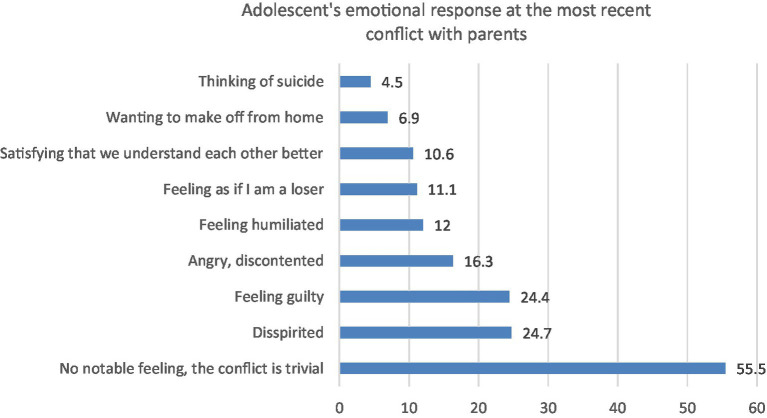
Adolescents’ emotional response at the most recent conflict with their parents.

Whereas adolescents’ behavioral responses seem not so alarming, report from adolescents shows that a part of them have negative feelings, as presented in Figure 3.

Beside 55.5% claim that the conflict is not so serious, so they have no notable feeling, 10.6% report that they feel satisfied because they and their parents develop better understanding of each other after the conflict. The remaining reports a quite alarming emotional response. Nearly 7% want to run away from home and 4.5% even think of committing suicide.

Whereas most of the parents behave quite supportively (67.2% of the mothers and 54.8% of the fathers of survey participants explained the situation/conflict to adolescents, and giving them advice), why do many of adolescents still have quite negative behavioral and emotional responses? Semi-structured interviews with adolescents show that adolescents tend to feel that their parents do not understand them and respect their needs.

‘My mom often says, “I gave birth to you, I understand you more than you do.” Anytime she says so, I do not want to talk with her anymore’ (12th grade student, girls, GPA at ‘Good’ level).

Even when parents give their adolescents reasonable advice, if this advice is not suitable to adolescents’ needs, the support of parents might create somehow negative feelings in adolescents. Following is one of the cases where parents provide support whereas teens feel disappointed. The communication between the adolescent and her parents, as narrated by the adolescent herself, somehow implies a parental invalidation. In this case, the parents love their daughter and care about every step in her progress. However, the way they take care of their daughter seems overprotective that the girl feels as if she is disabled to make decision for and take control of her own life. We hereafter quote a conversation between her and us to provide a better understanding of the girl’s perception and feeling towards her parents’ reaction.

Interviewer: Please tell me an example of your communication with parents which made you feel disappointed.

Interviewee: I want to study Psychology for my higher education program. First, my parents told me to let them think. A few days later, they asked me to have a talk with them and then they gave me a hundred reasons for why I should not study Psychology and why I should study Business instead. I cannot contradict, because all what they said was reasonable and full of evidence. It’s just… I want to study Psychology, not Business.

Interviewer: So, you disappointed with the fact that you must give up Psychology, or what?

Interviewee: With the communication. With my parents. About Psychology, I was not self-confident about my understanding of this discipline, so…, yes I did feel somewhat regretful for giving up but not too much disappointed.

Interviewer: I am trying to understand your situation. It took your parents some time to study both Psychology and Business before talking with you, it seems that they really care about you [the interviewee nodded]. At first you preferred Psychology, however the fact that you find their reasons reasonable, then you follow their advice, it seems that you are somewhat persuaded. What made you disappointed with that communication and your parents?

Interviewee: It was like my parents’ performance, and I am just an audience. I kept silent all the time. No room for me to talk. They gave me the reasons, and they made the decision for me. Like all the other times. Even though I know what they say is good for me, their decision will be better than mine because they have a lot of experience and knowledge. I cannot contradict anything they say. But I am still unsatisfied.

Interviewer: From what you said, I understand that your parents tend to impose their solution for your issues. Even though their solution may be the best one for you to select, the fact that you have no voice in the important milestones of your life makes you dissatisfied, because you are unable to manage your own life. Did I get you right?

Interviewee: Yes, exactly. It makes me feel I am unable to take control of my own life.

Results from the questionnaire survey is in the same line with qualitative data. One-way ANOVA test result showed that how adolescents feel after the conflict varied among different way in which their parents and them resolve the conflict. When being asked how they felt about the conflict (0 = completely unsatisfied; 3 = completely satisfied), results show that most of adolescents in the situation that parents impose their way of resolving the conflict on adolescent feel unsatisfied (mean score = 0.94 on a 4-point scale from 0–3, with a low standard deviation at 0.276). They also somewhat dissatisfy in the case that their parents ignore the conflict (mean score = 1.50, SD = 0.746). Adolescents are most satisfied with the situation where parents support their way (mean score = 2.56; SD = 0.577), followed by the situation where parents talk it over with them (mean score = 2.09; SD = 0.578). One-way ANOVA test result show that the differences between mean scores are statistically significant (*F*(3,706) = 178.670; *p* = 0.000).

### 3.3. Factors related and unrelated to adolescents’ frequency of having conflict with their parents

The following table summarizes associations between adolescents’ frequency of having conflict with parents and some personal and family factors. Where the independent variables are Likert scale or quantitative, we use Pearsons’r; and where they are categories, we use Chi-square analysis (See Table 4).

**Table 4:** The associations between parent-adolescent conflicts and some personal and family factors.

No association	Statistically significant associations
Adolescents’ sex; grade, and academic performance	Frequency of hanging out with friends (*r* = −0.130; *p* = 0.001)
The number of generations at home	Mothers’ education (*r* = 0.142; *p* = 0.000)
The number of children in the household	Father’s education (*r* = 0.143, *p* = 0.000)
Cozy and warm atmosphere at home; parents love each other	Parents’ conflicts with each other (0.152; *p* = 0.000)

Different from our expectations, adolescents’ sex (whether they are boys or girls), grade (whether they have just been enrolled to high-school – grade 10; or they are about to finish their high-school education and preparing for university entrance exam – grade 12); and academic performance (whether they obtained high or low GPA) have no statistically significant association with adolescents’ frequency of having conflict with their parents. Whether the family is nuclear (only parents and children) or extended (parents, children, grand-parents…) also has no relationship with adolescents’ frequency of having conflicts with their parents. In addition, how often adolescents have conflict with their parents was found unrelated to their feeling that their home is cozy and warm.

However, our study finds that Vietnamese adolescents’ frequency of having conflict with parents is statistically related to some parent- and friend-related factors, as following:

*Parents’ education level:* the higher education level the parent is at, the more frequently they have conflict with their adolescent child. This is true for both father and mother.

*Relationship between mother and father:* whereas the harmonious relationship between father and mother has no association with their frequency of having conflict with their teenage child, parents who often quarrel with each other tend to have more conflict with their teens.

*Friend network:* although Chi-square test shows no statistically significant relation between friend network and adolescents’ frequency of having conflict with parents, the distribution of having conflicts with parents over types of friend network worths considering, as presented in the following table:

As shown in Table 5, adolescents who have a healthy friend network (type 2 – having many friends, some of them are close friends; and type 3 – having not many friends, some of them are close friends) tend to have less conflict with their parents. Notably, there is a remarkably higher proportion of adolescents who are somewhat socially isolated (type 4 – having a few friends, not close with anyone) frequently have with parents (14.7%, three times that proportion among type 2; and about two times that proportion among type 1 and 3). On the contrary, the proportion of rarely having conflict with parents among type 2 was also higher than that proportion among type 1, 2, and 3 (50%, in comparison with 44.1%; 39.4%; 43.1%, respectively). The similar trend is also observed among adolescents who have many friends but are hardly close with anyone (type 1).

**Table 5:** Relationship between adolescents’ social relationship and their frequency of having conflict with parents.

	Adolescents’ frequency of having conflict with parents (%)				
	Rarely	Sometimes	Many times	Frequently	*N*
**Friend network** (n/a)					
Type 1	44.1	26.5	22.1	7.4	68
Type 2	39.4	31.6	24.2	4.8	393
Type 3	43.1	28.0	22.7	6.2	211
Type 4	50.0	26.5	8.8	14.7	34
Adolescents’ frequency of hanging out with friends (^*^)					
Nearly daily	33.3	22.7	30.7	13.3	75
Sometimes a week	42.1	26.3	25.8	5.7	209
Sometimes a month	39.5	34.7	21	4.8	248
Hardly	48.2	31.0	17.9	3.0	168

n/a: no association; (*) *p* < 0.001.

*Frequency of hanging out with friends:* the more frequently adolescents hang out with their friends, the more frequent conflict they have with their parents.

This study finds that adolescents who have very little time for hanging out with friends (almost no) tend to have less conflict with their parents, whereas those who spend too much time on hanging out with friends (nearly daily) have more conflicts with their parents.

## 4. Discussion

This exploratory study finds that Vietnamese parents and adolescents commonly have conflict over 09 issues: adolescents’ dressing and hair style; time spent with friends, academic performance, taking extra-classes; higher education orientation; self-study at home; money management; time spent on internet for entertainment purpose; and choosing friends. Among these issues, conflicts over adolescents’ spending time on the internet for entertainment purposes, self-study and academic performance occur most frequently among adolescents and their parents. It also adds that, beside the traditional concern for study, Vietnamese parents strongly worry that their adolescent spend too much time on the internet for entertainment.

Further, this study found that there was no difference between mothers and fathers in the trend of having conflict with their adolescent child over specific issues. Both mother and father have more conflicts over study, internet usage, and friend issues than other issues. This suggests that the Vietnamese mothers and fathers share similar concerns about their child.

This study found that Vietnamese parents, especially mothers, tend to act supportively when a conflict arose between them and their adolescents, as reported by adolescents themselves. Most of the parents would talk over the situation with their adolescents and provide them with advice. Even though some adolescents reported that their parents behave violently, the proportion is not too high, considering the fact that Vietnam is a culture which considers corporal punishment as a method of educating children (UNICEF, 2015). In addition, about 8% of Vietnamese adolescents reported that their fathers ignore the conflict. However, we believe this number does not mean these fathers neglect their adolescents. This finding may be better explained by the fact that in Vietnam it is the mother who plays the key role in taking care of and educating the children, hence in some trivial cases the father may completely entrust the mother with dealing with the parent–child conflict. This situation is quite popular in Asian cultures affected by Confucism (Keng and Soh, 2018).

However, the frequency of conflict between mother and adolescent is higher than between father and adolescent in all nine themes. In addition, no difference was found in the way mothers had conflict with their girls in comparison with boys. However, fathers have more conflicts with their boys than with their girls in most of the conflict themes, and these differences were statistically significant. We suppose that these differences may imply an intra-role conflict between fathers and sons, in which fathers tend to impose their expectations of being a man into their boys, and hence fathers have more conflicts with their boys than with their girls. However, we found no signals of the intra-role conflict between mother and girl. We suppose that this intra-role conflict between mother-and-girl may be neutralized by the fact that, in Vietnamese culture, mothers are often in charge for educating and taking care of their children (Teerawichitchainan et al., 2010). The more they undertake these education and care missions, the more chances they may have conflict with their adolescents, despite their adolescent’s sex.

Notably, the study finds that both behavioral and emotional response of Vietnamese adolescents when having conflict with their parents tend to be quite negative. They tend to act passively (e.g., either keeping silent and letting their parents talk; or obeying their parents’ arrangement even though they are unhappy with that). The rate of adolescents who have positive actions (e.g., trying to persuading their parents so that their parents understand their perspective and/or accept their solution), and the rate of those who have aggressive response (i.e., arguing aggressively with parents) are much lower than those who behave in a passive manner. Adolescents’ reports of their emotional responses even showed some alarming signals. Actually, half of them had no special feeling after their most recent conflict with their parents because the conflict was not serious; and even about one in ten reported a positive feeling that they were happy that the conflict resolution had helped them and their parents understand each other better. However, some reported quite alarming negative feelings such as feeling humiliated or feeling as if they were a loser. Remarkably, 4.5% of participants reported thought of committing suicide and 6.9% said they had wanted to run away from home. This result is in the same line with a recent report by UNICEF on the mental health of Vietnamese adolescent, which alarmed the risk of mental health issues and thought of committing suicide. Our in-depth interviews with Vietnamese adolescents suggested that the conflict between parents and adolescents itself was just stimulus. The key causes of these negative responses spring from the long-term relationship between parents and adolescents. In cases where the parent-adolescent conflicts were severe, our interviews with adolescents documented that the way their parents disrespect their autonomy, feeling and thought made them feel oppressed, impotent, and lonely. Remarkably, this situation also happened in families where parents love and over-protect their children. This study, hence, alarms some problems in Vietnamese parent-adolescent relationships which might results in adolescents’ poor mental health.

These findings are culturally understandable. Vietnam has been affected by Confucian ideology for a thousand of years; and Confucian concept of filial piety (“đạo Hiếu”) which requires children to unconditionally obey their parents (Bedford and Yeh, 2019). As the character “xiao is comprised of an upper component representing age and a lower component representing child” (Bedford and Yeh, 2019: 1), the concept of filial piety implies orderly social structure in which the parents are assigned the power to rules over the child. As a Vietnamese idiom goes, “Children must stay where their parents arrange” (cha me dat dau con ngoi day). This cultural belief lets Vietnamese parents tend to believe that they have the rights to decide things for their children. On the other hand, whereas adolescents have also been socialized this concept of filial piety, their profound exposure to new values and ideas such as children’s rights, autonomy, or individualism imported to Vietnam through mass media and cultural products (movies, novels, manga) creates generational differences between them and their adults. Though generation gap between parent and children occurs in many cultures (Kaufman, 1998; Williams and Nussbaum, 2001), it might occur more remarkably in Vietnam due to the socio-economic renovation in Vietnam since 1986 (Doi Moi). This turning point marked a radical change from a quite closed, bureaucratic, and subsidized economy to a market-oriented economy with a great effort to attract foreign investment in Vietnam. While Vietnamese parents in this study were children of the old economy, adolescent participants of this study were born when Vietnamese economy had been robustly developed and resulted in many social changes with a strong internationalization effort. Hence, though this study is discussing generational gap between adolescents and their parents in domestic Vietnamese families, the situation is quite identical to what the adolescents of migrated Vietnamese families in Poland are experiencing as found by Szymanska-Matusiewicz (2016): being “torn between two worlds” (p. 84). One world is the traditional values and norms, and the other one strongly affected by inter-national cultures especially Western cultures to which Vietnamese adolescents are daily exposed through various sources such as movies, novels, and especially social media platforms such as Facebook, Instagram, or Tik Tok. The warning point here, as this study discovered to some extent, is that this great difference gap between Vietnamese parents and adolescents, when combining with such an authoritarian parenting style, may result in the risk of mental health issue among adolescents such as low self-esteem, depression and anxiety (Fadlillah et al., 2020; Chen, 2022), and further childhood invalidation may in its turn may result in more severe issues like personality borderline disorder symptom (Keng and Soh, 2018).

Further, this study explores an interesting relation between teen friendship and adolescent-parent conflict. The more adolescents spend their time hanging out with friends, the more frequently they will have conflict with their parents. More frequent association with peers may broaden the generational differences between adolescents and their parents. This finding is in the same line with a study by Moon and Hofferth (2015), which found that the more time spent with peers and less time spent with parents increased adolescents’ externalizing behavior problems. Our study further found that adolescents having a healthy friend network tended to have less conflict with their parents. Adolescents having loose friend network tend to fall into two opposite categories: they might have either much more or, on the contrary, much less conflict with their parents. However, it should be noted that having less conflict with parents does not mean positive parent-teen conflict. This situation may reflect a harmonious relationship between parents and adolescents, but it may also imply a highly unharmonious relationship where adolescents hide their issues from their parents, or parents neglect their adolescents’ doings.

This study hence suggests that psychologists and social workers should pay more attention to the way parents and adolescents communicate with each other in daily life and how they perceive their relationships, the other’s expectations, and their own expectations of their parents/child. Developing more education programs for parents to better understand their adolescent child’s developmental needs and how parent–child relationship affects their child may be of great importance to Vietnamese families at this period.

## 5. Limitations and suggestions

This is an exploratory study aiming at discovering the relationship between parents and adolescents in a fast-developing country as Vietnam. It focuses on studying how Vietnamese parents and adolescent deal with their daily conflicts through the lens of adolescents. As an exploratory study, the study has captured key trends in parent-adolescent conflicts and the way they responded to their conflict, and further explored the associations between factors, both personal and family-related, and adolescent-parent conflict frequency. However, this study, with its exploratory nature, is unable to affirm some possibly significant associations between the loose friend network and the risk of parent–child conflict. Adolescent is the developmental period when peer relationship becomes critically important, therefore more study needs to be conducted to clarify how friend network and parent–child relationship is inter-related. This study also suggests that further qualitative and quantitative study should be conducted in order to clarify how parenting styles and parent-adolescent relationships may affect adolescents’ mental health and behavior problems in Vietnamese context. Whereas quantitative research helps clarify the correlations between parent-adolescent relationship and adolescents’ internalizing and externalizing problems, qualitative help provide more insightful understanding of how cultural beliefs and norms, family organization and division of labor, technology and the increasing participation of technology in personal living may contribute to parent-adolescent relationships.

## 6. Conclusion

This study focuses on investigating parent-adolescent conflicts in Vietnamese families, primarily from the perspectives of adolescents. A mixed-method approach involving in-depth interviews and questionnaire surveys was used to collect data for the study. The findings indicate that conflicts between Vietnamese parents and adolescents often arise regarding internet usage for entertainment purposes and academic matters. Furthermore, adolescents tend to experience more conflicts with their mothers than with their fathers. Although instances of parental aggression in response to conflicts were identified in the study, the majority of adolescents reported that their parents handled the conflicts in a supportive manner. However, Vietnamese adolescents likely to display passive behavioral responses. The study also identifies some warning signs of mental health issues in the emotional response of some adolescents such as suicidal thoughts. It is also found that more connection with peers significantly relates to the higher frequency of having conflict with parents. The study suggests that practitioners such as school social workers or counsellor should pay more attention to the impacts of parent–child conflict on adolescents’ emotion in particular and mental health in general, as well as trace back to the patterns of parenting and parent–child communication in contemporary Vietnamese families.

## Data availability statement

The raw data supporting the conclusions of this article will be made available by the authors, without undue reservation.

## Ethics statement

The studies involving humans were approved by Vietnam Institute for Family and Gender Studies. The studies were conducted in accordance with the local legislation and institutional requirements. Written informed consent for participation in this study was provided by the participants’ legal guardians/next of kin.

## Author contributions

THN designed the research, collected the data, run data analysis, and wrote the result session. TNN wrote introduction, research methods, discussion, limitations, and conclusion. All authors contributed to the article and approved the submitted version.
