# Supplementary material for: Parent-adolescent conflict: an exploration from the perspective of Vietnamese adolescents
Source: Front Psychol. 2023 Nov 7;14:1243494. doi: 10.3389/fpsyg.2023.1243494 (PMC10661886; doi:10.3389/fpsyg.2023.1243494)
Supplement: Supplementary file 1 [file Data_Sheet_1.docx]

Mã số phiếu:………………………….

Mã Trường học:………………………

| Mã lớp: ……………............................ |
| --- |

**BẢNG HỎI**

Chào em!

Trước hết, cám ơn em vì đã cùng tham gia cuộc khảo sát này!

Trên tay em là bảng hỏi phục vụ cho nghiên cứu về chủ đề Mối quan hệ cha mẹ và con cái độ tuổi THPT. Gửi đến em bảng hỏi này, chúng tôi mong muốn nhận được những thông tin, chia sẻ của các em về mối quan hệ với cha mẹ, về đời sống tinh thần của em. Mong em đừng nghĩ Liệu mình trả lời thế này là nên hay không nên, là tốt hay không tốt..Điều quan trọng duy nhất là **câu trả lời chân thật của chính em.** Và không một ai trong trường hay gia đình biết em trả lời như thế nào, và không ai được xem phần trả lời của em, trừ các nghiên cứu viên thuộc Viện Nghiên cứu Gia đình và Giới.

Mặc dù đây không phải bài thi, nhưng rất mong em sẽ trả lời chân thật và càng nhanh càng tốt. **Em vui lòng không bỏ trống các câu hỏi vì mọi thông tin đều có ý nghĩa rất lớn với nghiên cứu.**

Một lần nữa, trân trọng cảm ơn sự cộng tác của các em!

ThS. Nguyễn Thị Hồng Hạnh

Phòng Nghiên cứu Các vấn đề Xã Hội

Viện NC Gia đình và Giới, Viện HLKHXH Việt Nam

SĐT Liên hệ: 0902090005

Email: hanhnguyen.dnl@gmail.com

## PHẦN A: THÔNG TIN CHUNG

***Xin em vui lòng cho biết một số thông tin sau, hãy khoanh tròn vào phương án đúng với em:***

| **A1. Em là:** | 1. Nam | 1. Nữ | 1. LGBT |
| --- | --- | --- | --- |
| **A2. Em học lớp:** | 1. Lớp 10 | 1. Lớp 11 | 1. Lớp 12 |

**A3. Em vui lòng cho biết hoàn cảnh gia đình mình hiện nay:**

1. Em vẫn sống với cả bố và mẹ
2. Em chỉ sống với bố hoặc mẹ
3. Em sống với bố và mẹ kế
4. Em sống với mẹ và bố dượng

**A4. Trong gia đình, em có mấy anh chị em ( tính cả em**) :………

**A5. Em là người con thứ mấy trong gia đình?:** ………

**A6. Gia đình em có mấy thế hệ (tính cả em):……..** thế hệ

## B. VIỆC HỌC TẬP

**B1. Xin em cho biết điểm tổng kết của mình trong học kỳ vừa qua?**

| 1. Dưới 5,9 2. Từ 6,0 đến 6,9 | 1. Từ 7,0 đến 7,9 2. Từ 8,0 trở lên |
| --- | --- |

**B2. Hạnh kiểm trong Học kỳ qua của em là:**

| 1. Hạnh kiểm trung bình trở xuống | 1. Hạnh kiểm Khá | 1. Hạnh kiểm tốt |
| --- | --- | --- |

**B3. Ở trường lớp, em có đảm nhiệm vai trò nào dưới đây không?(**em có thể chọn nhiều phương án)

| 1. Cán bộ lớp   (lớp trưởng, lớp phó, tổ trưởng, tổ phó)   1. Thư ký lớp 2. Cán bộ Đoàn | 1. Cờ đỏ 2. Thành viên BCH Đoàn trường 3. Thành viên CLB trường 4. Không đảm nhiệm vai trò nào nêu trên |
| --- | --- |

**B4.** **Trong giai đoạn đi học (không tính nghỉ hè), trung bình một tuần em dành bao nhiêu thời gian để học tập**?( ví dụ: tuần em học thêm 5 ca, em nhân 5 với khoảng thời gian mỗi ca, em có thể phép nhân, đề tài sẽ tự điền kết quả)

| 1. Học thêm/ Học gia sư: ……… tiếng 2. Học năng khiếu: ……… tiếng | 1. Học chính thức ở trường: ……… tiếng 2. Tự học tại nhà:……… tiếng |
| --- | --- |

**B4.1.Với em, việc học thêm như vậy là vừa đủ hay bị ít/ nhiều?**

| 1. Học ít | 1. Học vừa đủ | 1. Học nhiều | 99. KAD( nếu em không học thêm) |
| --- | --- | --- | --- |

**B4.2 Với em, việc học năng khiếu như vậy là vừa đủ hay bị ít/ nhiều?**

| 1. Học ít | 1. Học vừa đủ | 1. Học nhiều | 99. KAD( nếu em không học năng khiếu) |
| --- | --- | --- | --- |

**B5.** **Nhìn chung, ai quyết định việc học thêm của em? Em khoanh tròn vào ô số tương ứng với lựa chọn của mình:**

|  | **Người quyết định việc học thêm/học gia sư/ học năng khiếu** | | | | |
| --- | --- | --- | --- | --- | --- |
|  | **Em tự quyết định hoàn toàn** | **Em quyết định là chính, nhưng có hỏi ý kiến bố mẹ** | **Em và bố mẹ cùng bàn bạc và quyết định** | **Bố mẹ quyết định nhưng có hỏi ý kiến em** | **Bố mẹ hoàn toàn quyết định** |
| Việc học thêm/học gia sư các môn chính khoá | 1 | 2 | 3 | 4 | 5 |
| Việc học năng khiếu | 1 | 2 | 3 | 4 | 5 |

**B6. Khi ở nhà, bố có hay nhắc nhở em chuyện học tập không**? 88. KAD- nếu em không ở cùng bố

| 1. Không nhắc nhở | 1. Ít nhắc nhở | 1. Thỉnh thoảng nhắc nhở | 1. Thường xuyên nhắc nhở |
| --- | --- | --- | --- |

**B7. Khi ở nhà, mẹ có hay nhắc nhở em chuyện học tập không**?88. KAD- nếu em không ở cùng mẹ

| 1. Không nhắc nhở | 1. Ít nhắc nhở | 1. Thỉnh thoảng nhắc nhở | 1. Thường xuyên nhắc nhở |
| --- | --- | --- | --- |

**B8. Em có hay chia sẻ với bố các khó khăn trong học tập không?** 88. KAD- nếu em không ở cùng bố

| 1. Không chia sẻ | 1. Ít chia sẻ | 1. Thỉnh thoảng chia sẻ | 1. Thường xuyên chia sẻ |
| --- | --- | --- | --- |

**B9. Em có hay chia sẻ với mẹ các khó khăn trong học tập không?**88. KAD- nếu em không ở cùng mẹ

| 1. Không chia sẻ | 1. Ít chia sẻ | 1. Thỉnh thoảng chia sẻ | 1. Thường xuyên chia sẻ |
| --- | --- | --- | --- |

**B10. Bố mẹ có ép em phải học kể cả khi em mệt không?**

| 1. Không bao giờ | 1. Có, Ít khi | 1. Có, Thỉnh thoảng | 1. Có, Thường xuyên |
| --- | --- | --- | --- |

**B11. Em có bị bố mẹ gây áp lực về việc đạt điểm cao không?**

| 1. Không bao giờ | 1. Có, Ít khi | 1. Có, Thỉnh thoảng | 1. Có, Thường xuyên |
| --- | --- | --- | --- |

**B12. Xin em cho biết, bố mẹ tham gia như thế nào vào việc chọn trường/lớp/ban học ( ban A, ban D) của em?**

1. Bố/mẹ quyết định hoàn toàn
2. Bố/mẹ quyết định có hỏi ý kiến em
3. Bố/mẹ và em cùng quyết đinh
4. Em quyết định có hỏi ý kiến Bố/mẹ
5. Em hoàn toàn quyết định

**B13. Khi bố/mẹ đóng vai trò quyết định chính trong việc chọn trường lớp của em, em có** **cảm nhận như thế nào?**

| 0 | 1 | 2 | 3 |
| --- | --- | --- | --- |
| Em thấy điều đó là hợp lý và tốt cho em | Em thấy không vấn đề gì | Em thấy hơi khó chịu | Em thấy cực kì  khó chịu |

# PHẦN C: SỬ DỤNG THỜI GIAN RỖI

**C1.Nếu hiểu Thời gian rỗi là khoảng thời gian em không phải đi học, không phải làm bài tập, cũng không phải làm các công việc bố mẹ/gia đình giao, mà là thời gian để nghỉ ngơi giải trí** *( không tính thời gian ngủ****)* thì trung bình, vào những ngày đi học,**

**em có bao nhiêu thời gian rảnh rỗi?.............** tiếng/01 ngày

**C2.Trung bình, vào ngày nghỉ cuối tuần, em có bao nhiêu thời gian rảnh rỗi?..............** tiếng

**C3.Em thường làm gì khi rảnh rỗi ?-** em hãy khoanh tròn 01 phương án phổ biến nhất.

| Vào những ngày đi học | Vào những ngày nghỉ cuối tuấn |
| --- | --- |
| 1. Gặp gỡ, giao lưu với các bạn *(đi chơi, đi trà chanh, trà sữa, đi dạo, sang nhà nhau chơi…..)* 2. Làm các hoạt động cá nhân một mình *( online, chơi game, đọc sách báo truyện)* 3. Tham gia các hoạt động giải trí cùng bố/mẹ | 1. Gặp gỡ, giao lưu với các bạn *(đi chơi, đi trà chanh, trà sữa, đi dạo, sang nhà nhau chơi…..)* 2. Làm các hoạt động cá nhân một mình *( online, chơi game, đọc sách báo truyện)* 3. Tham gia các hoạt động giải trí cùng bố/mẹ |

**C4.Em thấy khoảng thời gian rỗi của mình là ít, vừa đủ hay nhiều?**

| 1 điểm | 2 điểm | 3 điểm |
| --- | --- | --- |
| Em có ít thời gian rỗi | Em có vừa đủ thời gian rỗi | Em có nhiều thời gian rỗi |

**C5.** **Vào những lúc rảnh rỗi, em có được toàn quyền làm điều mình thích không?**

1. Em được hoàn toàn tự do làm điều mình muốn
2. Em được làm điều mình muốn, nhưng bố mẹ vẫn can thiệp
3. Em cảm thấy không được tự do lắm, bố mẹ vẫn can thiệp nhiều.
4. Bố mẹ can thiệp chặt chẽ vào khoảng thời gian riêng tư của em.

**C6. Em có các thiết bị dưới đây của riêng mình không?** (Em có thể chọn nhiều phương án)

1. Điện thoại di động (chỉ có chức năng nghe- gọi)
2. Điện thoại di động thông minh (kết nối được Internet)
3. Máy tính xách tay
4. Máy tính để bàn
5. Máy tính bảng
6. Sách điện tử
7. Đồng hồ đeo tay thông minh

**C7. Khi ở nhà, em có kết nối mạng Internet không?** 1. Có. 2. Không

**C8. Khi ở nhà, em có được tự do sử dụng Internet theo ý muốn không?**

| 0 | 1 | 2 | 3 | 4 |
| --- | --- | --- | --- | --- |
| Không tự do | Ít tự do | Tự do | Khá tự do | Rất tự do |
|  |  |  |  |  |

**C9 Em thường sử dụng Internet để làm gì nhiều nhất? (chỉ chọn MỘT phương án)**

1. Phục vụ việc học tập
2. Giao lưu với bạn bè qua mạng xã hội: nhắn tin, viết thư, comment facebook, chat..
3. Giải trí: xem phim, nghe nhạc, chơi game..
4. Tìm hiểu vấn đề mình quan tâm (thời sự, hội hoạ, âm nhạc…)
5. Khác ( em ghi rõ):……………………………

## D. HOẠT ĐỘNG CHI TIÊU

**D1. Em có khoản tiền tiêu vặt của riêng mình không?** 1. **Có** 🡪 câu D2 2. **Không** 🡪 chuyển câu D5

**D2. Em thấy khoản tiền tiêu vặt đó có đủ cho nhu cầu tiêu dùng của em không**?

| 1. Rất thiếu | 1. Hơi thiếu | 1. Đủ | 1. Thừa |
| --- | --- | --- | --- |

**D3. Trong 01 năm qua, đối với tiền tiêu vặt của mình, em có được quyền quyết định trong việc chi tiêu của mình không?**

1. Em hoàn toàn tự quyết định( *chuyển đến câu D7*)
2. Em quyết định là chính, bố mẹ chỉ tham gia 1 phần
3. Em với bố mẹ cùng bàn bạc và quyết định việc chi tiêu
4. Bố mẹ quyết định là chính, em chỉ tham gia 1 phần
5. Em không có quyền quyết định việc chi tiêu.

**D4. Nếu bố mẹ có tham gia vào việc chi tiêu của em, em có thấy hợp lý không?**

| 1. Hoàn toàn không hợp lý | 1. Hợp lý | 1. Rất hợp lý |
| --- | --- | --- |

**D5. Nếu cần thêm tiền tiêu, em có thấy thoải mái khi xin bố mẹ không?**

| 0.Hoàn toàn không thoải mái | 1. Không thoải mái | 2. Thoải mái | 3. Rất thoải mái |
| --- | --- | --- | --- |

**D6. Em có nghĩ ở tuổi mình, học sinh được quyền cầm và tự quyết định việc chi tiêu với số tiền mình có/mình được cho không?**

| 0 | 1 | 2 | 3 |
| --- | --- | --- | --- |
| Hoàn toàn không đồng ý | Không đồng ý | Đồng ý | Hoàn toàn đồng ý |

**D7. Trong 01 năm qua, em có đi làm thêm không (tính cả việc làm trong nhà được bố mẹ trả công)?**

1. Có, em làm thêm và nhận lương( chuyển đến D9)
2. Có, em làm thêm nhưng không có lương ( công việc tình nguyện..)( chuyển đến D9)
3. Không

**D8. Em có thể cho biết lí do em không đi làm thêm?**

1. Em không muốn đi làm thêm ( chuyển đến phần E)
2. Bố mẹ không cho em đi làm thêm ( chuyển đến D11)
3. Khác, em vui lòng ghi rõ:…………………………

**D9. Vì sao em quyết định đi làm thêm?( em có thể chọn nhiều phương án)**

| 1. Có thêm tiền tiêu 2. Có cơ hội giao lưu nhiều hơn 3. Không muốn ở nhà nhiều 4. Tăng cường kỹ năng sống, kỹ năng giao tiếp | 1. Em đi làm cùng bạn cho vui 2. Bố mẹ bắt em đi làm 3. Khác: em vui lòng ghi rõ………………….. |
| --- | --- |

**D10**. **Bố mẹ ủng hộ em đi làm thêm ở mức độ nào?**

| 1. Bố mẹ **không** ủng hộ chút nào | 1. Bố mẹ ủng hộ nhưng không thoải mái lắm | 1. Bố mẹ hoàn toàn ủng hộ cho em đi làm |
| --- | --- | --- |

**D11**. **Em có biết vì sao bố/mẹ không cho hoặc không ủng hộ em đi làm thêm?(Em có thể chọn nhiều phương án)**

1. Bố/mẹ lo ảnh hưởng đến học tập
2. Bố/mẹ lo ảnh hưởng đến sức khỏe
3. Bố/mẹ sợ em giao du với người xấu
4. Bố/mẹ sợ mất thể diện
5. Khác- em vui lòng ghi rõ: ………………………………………………

## E. QUAN HỆ BẠN BÈ

**E1. Nếu hiểu bạn thân là người mà em tin tưởng, thường xuyên liên lạc và có thể chia sẻ các suy nghĩ và cảm xúc thầm kín của mình, nhận định nào dưới đây mô tả đúng nhất mối quan hệ bạn bè của em**

1. Em có rất nhiều bạn, nhưng hầu như không có bạn thân
2. Em có rất nhiều bạn, một số trong đó là bạn thân
3. Em có ít bạn, nhưng hầu hết là bạn thân
4. Em có ít bạn, và cũng không thân với ai
5. Em gần như không chơi với ai

**E2. Trong năm vừa qua,** **Em có hay đi chơi cùng các bạn không?**( đến nhà nhau chơi, đi dạo, đi cà phê trà chanh, đi chơi game…)

| 1. Hàng ngày | 1. Vài lần một tuần | 1. Vài lần một tháng | 1. Một năm vài lần | 1. Gần như không |
| --- | --- | --- | --- | --- |

**E3 Trong học kỳ vừa qua, bố/mẹ có thường xuyên ngăn cản em đi chơi với bạn không?**

1. Có, thường xuyên
2. Có, thỉnh thoảng
3. Có, ít khi
4. Không bao giờ

**E4. Ngoài giờ đi học, em thường liên hệ với các bạn của mình như thế nào? Hãy chọn 1 cách liên lạc mà em hay dùng nhất:**

1. Gặp mặt trực tiếp ( đến nhà nhau chơi, đi chơi cùng nhau…)
2. Gọi điện thoại hoặc gửi tin nhắn điện thoại
3. Qua mạng xã hội ( facebook, zalo, tiktok…)
4. Em gần như không liên lạc với các bạn
5. Khác…………………….

**E5. Bố/mẹ có biết rõ về bạn thân của em không?**

| 0.Hoàn toàn không biết | 1. Biết một ít | 2.Biết khá rõ | 3.Biết rõ |
| --- | --- | --- | --- |

**E6. Em đánh giá mức độ can thiệp của bố mẹ vào việc lựa chọn bạn bè của mình như thế nào?**

1. Bố/mẹ không can thiệp ( em chuyển sang câu E8)
2. Bố/mẹ can thiệp ít
3. Bố/mẹ khá can thiệp nhưng vẫn để em tham gia quyết định
4. Bố/mẹ can thiệp hoàn toàn

**E7. Nếu bố/mẹ có can thiệp vào chuyện lựa chọn bạn bè của em, thì em có** **cảm nhận gì?**

| 0 | 1 | 2 | 3 |
| --- | --- | --- | --- |
| Em thấy điều đó là hợp lý và tốt cho em | Em thấy không có vấn đề gì | Em thấy hơi khó chịu | Em thấy cực kì  khó chịu |

**E8. Em có hay chia sẻ với bố về các mối quan hệ bạn bè của mình không?** 88. KAD- nếu em không ở cùng bố

| 1. Không chia sẻ | 1. Ít chia sẻ | 1. Thỉnh thoảng chia sẻ | 1. Thường xuyên chia sẻ |
| --- | --- | --- | --- |

**E9. Em có hay chia sẻ với mẹ về các mối quan hệ bạn bè của mình không?** 88. KAD- nếu em không ở cùng mẹ

| 1. Không chia sẻ | 1. Ít chia sẻ | 1. Thỉnh thoảng chia sẻ | 1. Thường xuyên chia sẻ |
| --- | --- | --- | --- |

# F. QUAN HỆ GIỮA EM VÀ BỐ MẸ

**F1. Khi có tâm sự, em có hay chia sẻ với bố không?** *88. KAD- nếu em không ở cùng bố*

| 1. Không chia sẻ | 1. Ít chia sẻ | 1. Thỉnh thoảng chia sẻ | 1. Thường xuyên chia sẻ |
| --- | --- | --- | --- |

**F2. Khi có tâm sự, em có hay chia sẻ với mẹ không?** *88. KAD- nếu em không ở cùng mẹ*

| 1. Không chia sẻ | 1. Ít chia sẻ | 1. Thỉnh thoảng chia sẻ | 1. Thường xuyên chia sẻ |
| --- | --- | --- | --- |

**F3. Giữa bố và mẹ, ai thường xuyên nhắc nhở, sát sao với em trong cuộc sống hàng ngày hơn?**

| 1. Bố | 1. Mẹ | 88. KAD *nếu em không ở cùng cả bố và mẹ* |
| --- | --- | --- |

**F4. Giữa bố và mẹ, em cảm thấy gần gũi về mặt tình cảm với ai nhiều hơn?**

| 1. Bố | 1. Mẹ | 88. KAD *nếu em không ở cùng cả bố và mẹ* |
| --- | --- | --- |

**F5. Dưới đây là các nhận định về mối quan hệ giữa bố và mẹ em , em đồng ý ở mức độ nào với các nhận định này?**

|  | Hoàn toàn không đồng ý | Không đồng ý lắm | Đồng ý | Khá đồng ý | Hoàn toàn đồng ý | KAD- nếu em không ở cùng cả bố và mẹ |
| --- | --- | --- | --- | --- | --- | --- |
| 1. Gia đình em hoà thuận | 0 | 1 | 2 | 3 | 4 | 88 |
| 1. Bố mẹ em thường xuyên tranh luận to tiếng | 0 | 1 | 2 | 3 | 4 | 88 |
| 1. Bố mẹ em thương yêu nhau | 0 | 1 | 2 | 3 | 4 | 88 |
| 1. Bố mẹ thường xuyên tâm sự, trò chuyện với nhau | 0 | 1 | 2 | 3 | 4 | 88 |
| 1. Trong gia đình em, mẹ luôn nghe lời bố | 0 | 1 | 2 | 3 | 4 | 88 |
| 1. Bố mẹ em thường xuyên đánh chửi nhau | 0 | 1 | 2 | 3 | 4 | 88 |

**F6.Em đồng ý như thế nào với các nhận định dưới đây?**

|  | **Hoàn toàn không đồng ý** | **Không đồng ý lắm** | **Đồng ý** | **Khá đồng ý** | **Hoàn toàn đồng ý** |
| --- | --- | --- | --- | --- | --- |
| 1. Nghĩa vụ của con cái là nghe lời cha mẹ hoàn toàn | 0 | 1 | 2 | 3 | 4 |
| 1. Những điều bố mẹ xếp đặt cho con cái chưa chắc đã là điều con muốn | 0 | 1 | 2 | 3 | 4 |
| 1. Nhìn chung, khi quan điểm giữa con cái và bố mẹ khác nhau, con cái có quyền nói lên ý kiến của mình | 0 | 1 | 2 | 3 | 4 |
| 1. Con cái cần có được quyết định hoàn toàn các việc cá nhân của mình | 0 | 1 | 2 | 3 | 4 |
| 1. Con cái có thể cãi lại bố mẹ trong một vài trường hợp đặc biệt | 0 | 1 | 2 | 3 | 4 |

**F7.Trong mối quan hệ với cha mẹ, em đồng ý như thế nào với các nhận định dưới đây?**

|  | **Hoàn toàn không đồng ý** | **Không đồng ý lắm** | **Đồng ý** | **Khá đồng ý** | **Hoàn toàn đồng ý** | **KAD- nếu em không ở cùng cả bố và mẹ** |
| --- | --- | --- | --- | --- | --- | --- |
| 1. Em coi bố mẹ là những người bạn của mình | 0 | 1 | 2 | 3 | 4 | 88 |
| 1. Bố mẹ thường lắng nghe và tôn trọng ý kiến của em | 0 | 1 | 2 | 3 | 4 | 88 |
| 1. Bố mẹ chưa hiểu rõ về tính cách của em | 0 | 1 | 2 | 3 | 4 | 88 |
| 1. Bố mẹ hay áp đặt cho cuộc sống của em | 0 | 1 | 2 | 3 | 4 | 88 |
| 1. Em chỉ cần phản đối ý kiến của bố mẹ là bị gọi là “hỗn”, “ trứng khôn hơn vịt”… | 0 | 1 | 2 | 3 | 4 | 88 |
| 1. Nghe bố mẹ nhắc nhở, khuyên bảo nhiều khiến em mệt mỏi | 0 | 1 | 2 | 3 | 4 | 88 |

**F8.** **Trong học kỳ vừa qua, em và bố mẹ có thường xảy ra mâu thuẫn ở các lĩnh vực này không?**

|  | **Với bố** | | | | | **Với mẹ** | | | | |
| --- | --- | --- | --- | --- | --- | --- | --- | --- | --- | --- |
|  | Hàng ngày | Vài lần một tuần | Vài lần một tháng | Không có mâu thuẫn | KAD: nếu em không ở cùng bố | Hàng ngày | Vài lần một tuần | Vài lần một tháng | Không có mâu thuẫn | KAD: nếu em không ở cùng mẹ |
| 1.Chuyện ăn mặc, kiểu tóc của em | 1 | 2 | 3 | 4 | 88 | 1 | 2 | 3 | 4 | 88 |
| 2.Thời gian em giao lưu với bạn bè | 1 | 2 | 3 | 4 | 88 | 1 | 2 | 3 | 4 | 88 |
| 3.Điểm số học tập | 1 | 2 | 3 | 4 | 88 | 1 | 2 | 3 | 4 | 88 |
| 4.Việc đi học thêm của em | 1 | 2 | 3 | 4 | 88 | 1 | 2 | 3 | 4 | 88 |
| 5.Việc chọn trường lớp | 1 | 2 | 3 | 4 | 88 | 1 | 2 | 3 | 4 | 88 |
| 6.Việc học ở nhà của em | 1 | 2 | 3 | 4 | 88 | 1 | 2 | 3 | 4 | 88 |
| 7.Việc sở hữu và sử dụng tiền của em | 1 | 2 | 3 | 4 | 88 | 1 | 2 | 3 | 4 | 88 |
| 8.Thời gian sử dụng Internet/thiết bị công nghệ của em | 1 | 2 | 3 | 4 | 88 | 1 | 2 | 3 | 4 | 88 |
| 9.Việc chọn bạn của em | 1 | 2 | 3 | 4 | 88 | 1 | 2 | 3 | 4 | 88 |

**F9.** **Trong học kỳ vừa qua, em đánh giá mâu thuẫn giữa em và bố mẹ thường diễn ra ở mức độ nào? Em có thể hiểu mức độ nghiêm trọng là mức độ em cảm thấy tổn thương trong lần mâu thuẫn đó**

|  | Với bố | | | | Với mẹ | | |  |  |  |
| --- | --- | --- | --- | --- | --- | --- | --- | --- | --- | --- |
|  | Không nghiêm trọng | Bình thường | Nghiêm trọng | KAD nếu em không ở với bố | Không nghiêm trọng | Bình thường | Nghiêm trọng | KAD nếu em không ở với mẹ |  |  |
| 1.Chuyện ăn mặc, kiểu tóc của em | 0 | 1 | 2 | 88 | 0 | 1 | 2 | 88 |  |  |
| 2.Thời gian em giao lưu với bạn bè | 0 | 1 | 2 | 88 | 0 | 1 | 2 | 88 |  |  |
| 3.Điểm số ở trường | 0 | 1 | 2 | 88 | 0 | 1 | 2 | 88 |  |  |
| 4.Việc đi học thêm của em | 0 | 1 | 2 | 88 | 0 | 1 | 2 | 88 |  |  |
| 5.Việc chọn trường lớp | 0 | 1 | 2 | 88 | 0 | 1 | 2 | 88 |  |  |
| 6.Việc học ở nhà của em | 0 | 1 | 2 | 88 | 0 | 1 | 2 | 88 |  |  |
| 7.Việc sở hữu và sử dụng tiền của em | 0 | 1 | 2 | 88 | 0 | 1 | 2 | 88 | 4 | 88 |
| 8.Thời gian sử dụng Internet/thiết bị công nghệ của em | 0 | 1 | 2 | 88 | 0 | 1 | 2 | 88 |  |  |
| 9.Việc chọn bạn của em | 0 | 1 | 2 | 88 | 0 | 1 | 2 | 88 |  |  |

***Trong số các chủ đề : Học tập, Quan hệ bạn bè, Sử dụng thiết bị công nghệ/Internet, Cách em sử dụng tiền… được đề cập bên trên, em hãy nhớ lại lần mâu thuẫn gần đây nhất giữa em và bố/mẹ liên quan dến chủ đề trên, và cho biết:***

**G1 . Mâu thuẫn đó là về chuyện gì:……………………………………… ……………………………**

**G2. Lần đó, em mâu thuẫn với** : 1. Bố/bố dượng 2. Mẹ/mẹ kế 3. Cả bố và mẹ

**G3 Em có thể vui lòng mô tả đôi chút về lần mâu thuẫn đó?........................................................**

**………………………………………………………………………………………………………….**

**G4 Em đánh giá mức độ mâu thuẫn của lần đó như thế nào?**

| 1. Không nghiêm trọng | 1. Ít nghiêm trọng | 1. Nghiêm trọng | 1. Rất nghiêm trọng |
| --- | --- | --- | --- |

**G5. Khi xảy ra mâu thuẫn, em đã phản ứng ra sao?( em có thể chọn nhiều phương án)**

1. Im lặng không nói gì
2. Tìm cách né tránh bố mẹ
3. Giải thích, trình bày nhẹ nhàng với bố mẹ
4. Nghe theo bố mẹ nhưng trong lòng không phục
5. Tranh luận gay gắt với bố mẹ
6. Cảm thấy mình sai và thấy hối lỗi với bố mẹ
7. Khác:…………………….

**G6.** **Khi xảy ra mâu thuẫn, bố mẹ em đã phản ứng như thế nào?**( em có thể chọn nhiều PA)

| **Phản ứng của bố** | **Phản ứng của mẹ** |
| --- | --- |
| 1. Nói chuyện, khuyên bảo nhẹ nhàng, giải thích đúng sai cho em 2. Phạt nghiêm khắc nhưng em thấy công bằng, hợp lý 3. Mắng chửi gay gắt, khiến em cảm thấy bị xúc phạm 4. Áp dụng hình phạt với em 5. Liên lạc với cô giáo, nhà trường 6. Đánh em 7. Không làm gì 8. Khác:…………………………… 9. KAD( khi em ko ở cùng bố/ bố không ở nhà trong thời điểm đó) | 1. Nói chuyện, khuyên bảo nhẹ nhàng, giải thích đúng sai cho em 2. Phạt nghiêm khắc nhưng em thấy công bằng, hợp lý 3. Mắng chửi gay gắt, khiến em cảm thấy bị xúc phạm 4. Áp dụng hình phạt với em 5. Liên lạc với cô giáo, nhà trường 6. Đánh em 7. Không làm gì 8. Khác:…………………………… 9. KAD( khi em ko ở cùng mẹ/ mẹ không ở nhà trong thời điểm đó) |

**G7**: **Em và bố mẹ đã mất bao lâu để trở lại bình thường:……………… ngày**

**G8.Em cảm thấy như thế nào khi chuyện đó xảy ra?( em có thể chọn nhiều phương án)**

1. Em thấy bình thường, chuyện này cũng nhỏ
2. Em cảm thấy giận dữ, bất mãn với bố mẹ
3. Em thấy chán nản
4. Em muốn chết đi cho rồi
5. Em muốn bỏ nhà đi thật xa
6. Em thấy bị xúc phạm
7. Em ước mình không phải con của bố mẹ
8. Em thấy bản thân mình thật kém cỏi
9. Em thấy mình có lỗi
10. Em và bố/mẹ hiểu và gần gũi với nhau hơn
11. Khác:……………………………………………………………………………………

**G9. Em và bố mẹ giải quyết mâu thuẫn đó như thế nào?**

1. Em nghe theo lời bố mẹ
2. Bố mẹ thuận theo ý em
3. Em và bố mẹ cùng đồng thuận giải quyết
4. Em và bố/mẹ nhờ người thứ ba phân xử
5. Em và bố/mẹ không đề cập tới chuyện đó nữa, lờ đi coi như chưa từng xảy ra

**G10. Em hài lòng như thế nào về cách giải quyết lần đó?**

| 0 | 1 | 2 | 3 |
| --- | --- | --- | --- |
| Hoàn toàn không hài lòng | Không hài lòng | Hài lòng | Hoàn toàn hài lòng |

**G11. Em và bố mẹ đã làm gì để hàn gắn sau lần mâu thuẫn đó**

Em đã………………………………………………………………………………………………

Bố mẹ đã……………………………………………………………………………………………

**Cuối cùng, xin em vui lòng cho biết một số thông tin về bố mẹ của mình nhé.**

|  | **Bố** | **Mẹ** |
| --- | --- | --- |
| G12.Tuổi  (nếu em nhớ năm sinh của Bố mẹ, em điền năm sinh. Nếu em nhớ tuổi/ khoảng tuổi của bố mẹ thì em điền vào dòng tuổi) | Tuổi:………… | Tuổi:………… |
|  | Năm sinh:………….. | Năm sinh:………….. |
| G13.Học vấn |  |  |
|  | 1. Cấp I | 1. Cấp I |
|  | 2. Cấp 2 | 2. Cấp 2 |
|  | 3. Cấp 3 | 3. Cấp 3 |
|  | 4. Cao đắng/ Đại học | 4. Cao đắng/Đại học |
|  | 5. Trên đại học | 5. Trên đại học |
|  | 6. Không biết | 6. Không biết |
| G14.Nghề nghiệp | 1.Làm cơ quan NN, công ty, doanh nghiệp | 1.Làm cơ quan NN, công ty, doanh nghiệp |
|  | 2.Công nhân, lao động tự do | 2.Công nhân, lao động tự do |
|  | 3.Buôn bán, kinh doanh, dịch vụ | 3.Buôn bán, kinh doanh, dịch vụ |
|  | 4.Nội trợ/Hưu trí/Không làm việc | 4.Nội trợ/Hưu trí/Không làm việc |
|  | 5.Khác | 5.Khác |
|  | 6.Không biết | 6.Không biết |

***Chân thành cám ơn em đã dành thời gian hỗ trợ nghiên cứu!***

SĐT của em (nếu có):………………………….

Em có nhu cầu nói chuyện thêm về chủ đề này không?( nếu có, tôi sẽ chủ động liên lạc với em)

| 1. Có | 1. Không |
| --- | --- |
